# In silico method for selecting residue pairs for single-molecule microscopy and spectroscopy

**DOI:** 10.1038/s41598-021-85003-0

**Published:** 2021-03-11

**Authors:** Hendrik R. Sikkema, Bert Poolman

**Affiliations:** Department of Biochemistry, Groningen Biomolecular Sciences and Biotechnology Institute & Zernike Institute for Advanced Materials, University of Groningen, Nijenborgh 4, 9747 AG Groningen, The Netherlands

**Keywords:** Biochemistry, Biological techniques, Chemical biology

## Abstract

Obtaining (dynamic) structure related information on proteins is key for understanding their function. Methods as single-molecule Förster Resonance Energy Transfer (smFRET) and Electron Paramagnetic Resonance (EPR) that measure distances between labeled residues to obtain dynamic information rely on selection of suitable residue pairs for chemical modification. Selection of pairs of amino acids, that show sufficient distance changes upon activity of the protein, can be a tedious process. Here we present an in silico approach that makes use of two or more structures (or structure models) to filter suitable residue pairs for FRET or EPR from all possible pairs within the protein. We apply the method for the study of the conformational dynamics of the substrate-binding domain of the osmoregulatory ATP-Binding Cassette transporter OpuA. This method speeds up the process of designing mutants, and because of its systematic nature, the chances of missing promising candidates are reduced.

## Introduction

Years of X-ray crystallography, NMR and more recently CryoEM^1,2^ have made available a wealth of structural data for (membrane) proteins. Even though all these techniques can give information on protein dynamics, for instance using caged compounds and free-electron lasers in serial crystallography^3^, or imaging under turnover conditions in CryoEM^4^, transient states and continuous dynamics are not readily obtained. Double electron–electron resonance (DEER) or Pulsed Electron–electron double resonance (PELDOR) can be used to probe distance changes upon changing conditions^5^ and single-molecule Förster Resonance Energy Transfer (smFRET) can be used to obtain single molecule dynamics of proteins and other macromolecular assemblies^6–10^. The latter two techniques make use of two labels that are introduced for instance by attaching them to cysteine residues via maleimide^11^ or methanethiosulfonate chemistry^12^ or introducing them as non-natural amino acids^13^. SmFRET even enables study of protein dynamics in vivo^12^. In vitro studies can provide a wealth of dynamics information, for instance when smFRET is applied to determine the dynamics of single-surface attached proteins upon addition of a ligand or e.g. photoactivation^10^.

There are a few difficulties in the application of the optical microscopy or electron spin resonance techniques. A key challenge is the selection of the labeling sites and the selectivity of the modifications. Both smFRET and EPR detect a change in distance between the labels, therefore naturally, the label should report a change in conformation. But even when they do, there are more restrictions. For instance, FRET typically occurs at spacings smaller than 8–10 nm, and the signal is the strongest when the distance is close to the Förster radius (R_0_), which is a property of the used FRET-pair^14^. On the same note, the larger the distance change, the larger the change in FRET signal, thus when selecting FRET pairs, one typically looks for pairs making large movements. Similarly, PELDOR is sensitive for distance changes in the range from 1.6 to 8 nm^5,15^. Furthermore, the site of labeling should be solvent-accessible, the label should be able to rotate freely to prevent anisotropic artifacts and the modification should not affect the functionality of the protein. In FRET, where a fluorescence donor and acceptor label are required, the labeling efficiency makes the procedure even more complicated. A typical labeling with donor (D) and acceptor (A) yields 25% of DD, 25% of AA and therefore only half of the particles are useful (25% of DA + 25% of AD). Alternating Laser EXcitation (ALEX), TIRF-based smFRET^16^ and Pulsed Interleaved Excitation (PIE) spectroscopy^17,18^ are techniques that allow FRET measurements corrected for the unwanted DD and AA populations.

All these restrictions make the selection of labeling sites challenging. Hand picking is tedious and one easily misses potentially useful sites because the approach is easily biased. We have developed a systematic in silico method that makes use of two or more protein structures that differ in conformation and applies distance and accessibility restraints to all possible residue pairs. If only one high-resolution structure is available, the combination with a carefully designed homology model of the other conformation should also work. Our method reduces the number of possibilities drastically and allows to focus on the biological restraints rather than the technical ones to obtain the best possible residue pairs. We tested the method by designing pairs for labelling in the substrate-binding domain (OpuAC) of the ABC transporter OpuA and performed single-molecule FRET measurements and functionality assays of the full-length protein complex.

## Results

### The ABC-transporter OpuA

The protein that we use to showcase the in silico approach is the osmoregulatory ABC transporter OpuA. Its substrate-binding protein (OpuAC) undergoes a conformational change upon binding of glycine betaine. Manually selected labelling positions are already available for this protein, which have been used in previous smFRET studies^10^. Even though the used labelling sites (V360C/N423C) report large differences in distance upon glycine betaine binding and do not affect the binding process, they affect the transfer of substrate from the SBD to the membrane domain of OpuA. In fact, the V360 and N423 are present in the lobes of OpuAC that interact with the transmembrane domain (TMD) of OpuA^19^. Therefore, we aimed to find new residue pairs for smFRET that do not interfere with the docking of OpuAC. We present a general procedure for selecting labelling sites based on a minimum of two protein structures. This method provides a screening of all possible residue pairs and allows smart filtering, prior to performing the actual experiments. The results can be inspected manually, for instance by using knowledge of the activity and structure of the entire protein complex. In our case we manually filtered out regions that would affect the interaction of the substrate-binding domain (here OpuAC) with the TMD of the OpuA complex^19^.

### In silico distance mapping

**Figure 1:**
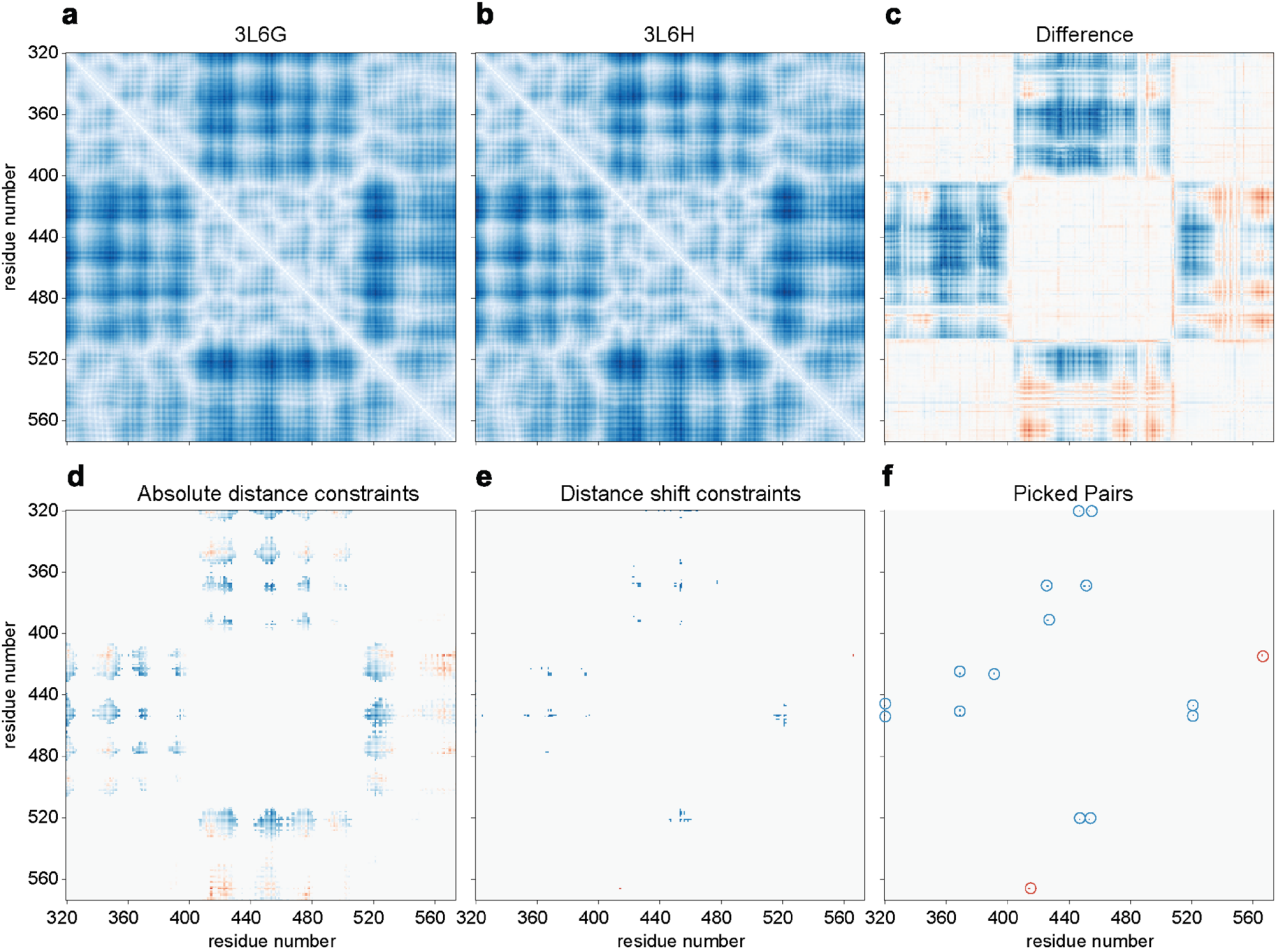
Maps, showing the distance between the centers of mass of the sidechains (C_alpha_ for glycine) of all possible residue pairs in the (**a**) Open conformation (PDB: 3L6G) and (**b**) Closed liganded conformation (PDB: 3L6H) of OpuAC. Panel (**c**) shows the distance change when transitioning between the two conformations; the orange color indicates a decreasing distance upon binding of glycine betaine to the open conformation; the blue color shows an increasing distance upon ligand binding. Panel (**d**) The same as panel **c** but now the pairs with an absolute distance larger than 80 Å and smaller than 40 Å are filtered out. Panel (**e**), the same as panel **d** but now pairs with a distance change smaller than 8 Å are filtered out. Panel (**f**), the same as panel **e** but now all residues that are less than 60% surface-exposed are filtered out; circles are drawn around pairs/clusters to increase visibility.

Crystal structures of OpuAC in the open (PDB: 3L6G) and ligand-bound closed (PDB: 3L6H) conformation^20^ were used as a starting point for the in silico distance mapping. In short, a distance map plots the distance between each possible pair of residues, in this case between the two centers of mass of the side chains of the amino acids (Cα in case of glycine). The center of mass of the sidechain was chosen, because it is closer to the site of labelling than Cα and it takes the direction of the sidechain into account; in the script (https://github.com/MembraneEnzymology/ResiduePairs) the center of mass is easily changed into Cα, if preferred. This (d_conformationA_ d_conformationB_) leads to a symmetrical (d_1,2_ = d_2,1_) area plot (Fig. 1a,b). Next, a distance change map is generated by subtracting the distance map of the second conformation (here the closed state of OpuAC) from the first one (open state) (Eq. 1). This difference map shows the distance shift for each residue pair upon the conformational change that is elicited by the binding of glycine betaine (Fig. 1c).

1 $${\text{d}}_{{{\text{conformationA}}}} - {\text{d}}_{{{\text{conformationB}}}} = {\text{d}}_{{{\text{conformationAB}}}}$$

### Filtering of the results

The three obtained maps (Fig. 1a–c) contain all possible pairs of residues, which can now be used to apply restraints in a mathematical way to the residue pairs. First, we select for distances within the predefined range (*e.g.* as set by the R_o_ value of the FRET pair), by discarding pairs with distances that are larger or smaller than two threshold values (Eqs. 2 and 3). The resulting pairs are shown in Fig. 1d.

2 $${\text{d}}_{{{\text{min}}}} < {\text{d}}_{{{\text{conformationA}}}} < {\text{d}}_{{{\text{max}}}}$$

3 $${\text{d}}_{{{\text{min}}}} < {\text{d}}_{{{\text{conformationB}}}} < {\text{d}}_{{{\text{max}}}}$$

We then establish a minimum threshold for the distance shift required for the smFRET measurements (Eq. 4). The resulting pairs are shown in Fig. 1e.

4 $${\text{d}}_{{{\text{conformationAB}}}} {\text{ > d}}_{{{\text{shift}} - {\text{threshold}}}}$$

Finally, the absolute accessible area is calculated using the DSSP program, which makes use of the structure of the protein to calculate properties as secondary structure, bond and torsion angles and water-exposed surface area^21^. Using solvent exposed residues is important to ensure accessibility for the probe to react but also to allow free rotation of the label. The total accessible area from the DSSP program is then divided by the theoretical total surface area for that residue (used values are the calculated surface area for the amino acid X in a Gly-X-Gly tripeptide from^22^, giving the relative surface accessibility (RSA). The amino acid pairs with a sufficient RSA in both conformations are kept (Fig. 1f), all others are discarded. All the defined thresholds can be adjusted to suit specific needs or to reduce the number of remaining pairs. Similarly, one could easily extend the filtering method based on secondary structure, as labelling of loop regions is typically favoured over structured areas. Secondary structure is also calculated by the DSSP program. A customizable script is available on GitHub: https://doi.org/10.5281/zenodo.4446814 or https://github.com/MembraneEnzymology/ResiduePairs.

**Figure 2:**
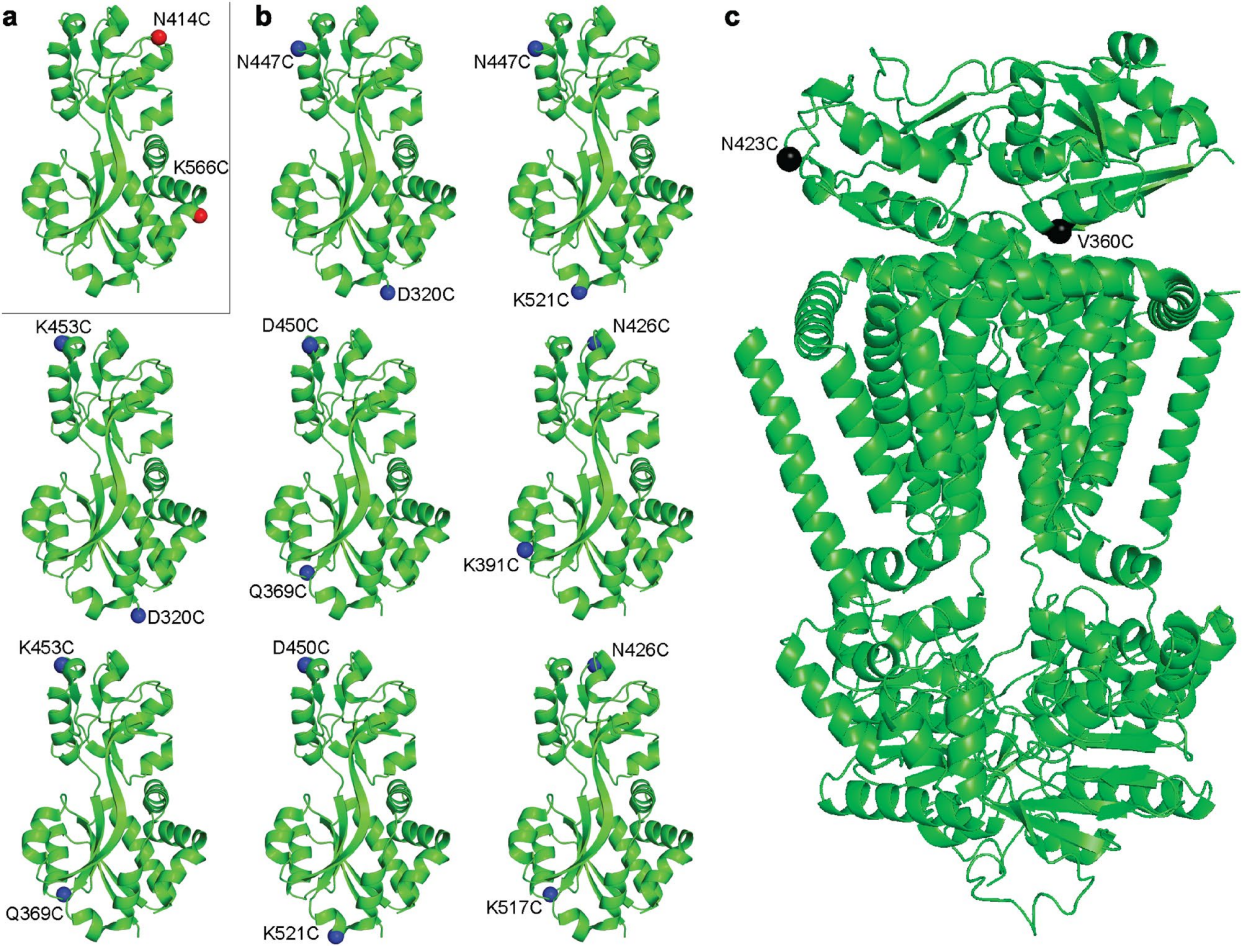
The 9 selected residue pairs shown in cartoon representation in OpuAC (PDB: 3L6G) (from left to right, top to bottom: N414C/K566C, D320C/N447C, N447C/K521C, D320C/K453C, Q369C/D450C, K391C/N426C, Q369C/K453C, D450C/K521C and N426C/K517C). The location of the amino acid pairs for dye labeling are shown as spheres. (**a**) Red spheres indicate a decreasing distance upon glycine betaine binding. (**b**) Blue spheres indicate an increasing distance upon binding. (**c**) Shows the positions of the Cys residues (V360C/N423C) previously used for labeling of OpuAC in the full-length protein (PDB: 7AHD), see Sikkema et al^19^ for the full-length structure of OpuA; labeling of these residues affects the docking of the SBDs and therefore they cannot be used for smFRET studies of full-length OpuA.

In the case of OpuAC we used the following thresholds: d_min_ = 40 Å, d_max_ = 80 Å, d_shift-threshold_ = 8 Å, RSA = 60% to obtain 9 pairs, shown in Fig. 2a,b. These pairs were exported to PyMOL for manual inspection, where we aimed for pairs located on the sides of OpuAC that do not interfere with the docking of the substrate-binding domain in the full-transporter complex (Fig. 2c). We selected two pairs, one with a positive FRET change upon glycine betaine binding (D320C/K453C) and one with a negative FRET change upon binding (N414C/K566C). We also include a pair (T504C/K521C) with a positive FRET signal and a low relative surface accessibility (RSA = 8–11% for Thr504). The parameters of all three mutants plus the original mutant (V360C/N423C) are shown in Table 1.

**Table 1:** Distance and surface accessibility parameters for the original and newly selected mutants.

Residue pair	Δd_AB_	d_A_	d_B_	RSA_1B_	RSA_2B_	RSA_1A_	RSA_2A_
D320C/K453C	9.4	67.3	57.9	91.7	90.7	105.7	88.6
T504C/K521C	6.4	47.8	41.4	8.8	88.1	11.4	91.7
N414C/K566C	− 8.4	40.0	48.5	79.8	72.5	77.7	77.2
V360C/N423C	9.6	48.5	39.0	29.5	60.1	35.8	59.6

Δd_AB_ is Δd_conformationAB_ in Å, Δd_X_ is Δd_conformationX_ in Å, and RSA_nX_ is the relative surface accessibility of residue n in conformation X and %.

### Mutations in OpuAC, the substrate-binding domain of OpuA

**Figure 3:**
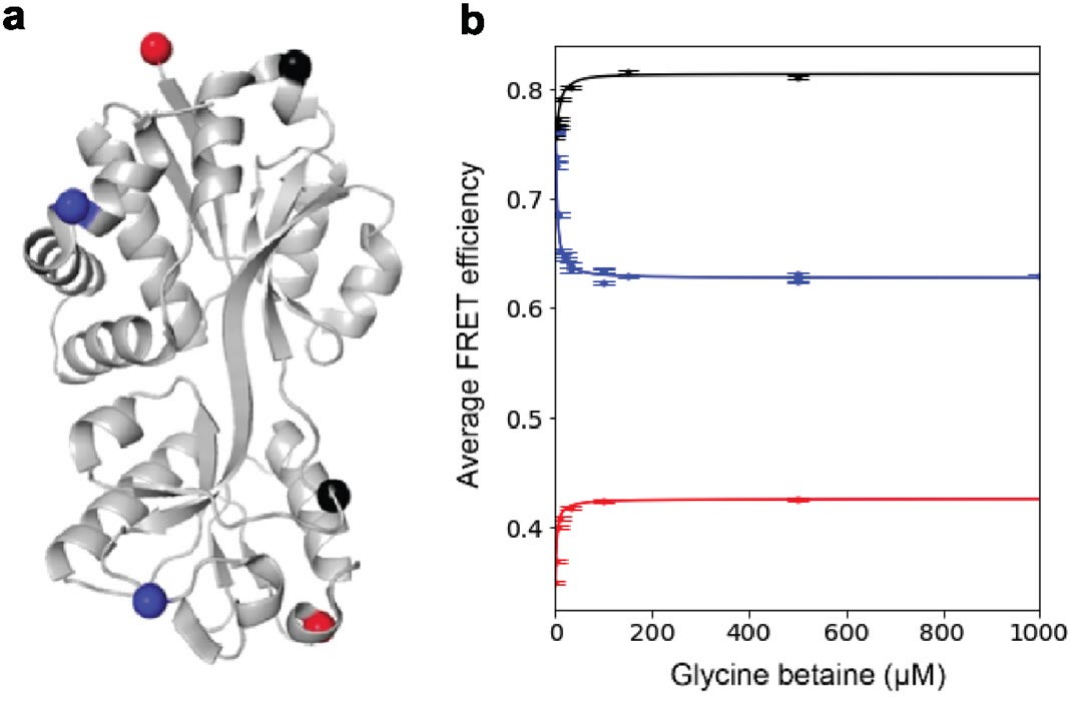
The three newly selected SBD mutation pairs that were constructed for OpuAC. (**a**) Structural representation (PDB:3L6G); red: (D320C/K453C), black: (T504C/K521C) and blue: (N414C/K566C). (**b**) FRET change upon binding of glycine betaine in the FRET pairs depicted in panel (**a**); the color coding is the same as in the structure; error bars represent the standard deviation obtained from the Gaussian fit in the smFRET data analysis.

The three newly selected mutants (Table 1) were first constructed in the SBD of OpuA, which were expressed as water-soluble proteins (named OpuAC) and purified to homogeneity. Glycine betaine titrations were performed to assure normal function of the mutant and fluorophore-labelled proteins. OpuAC with double cysteines were labelled with the fluorescence donor (Alexa555) and acceptor (Alexa647), using maleimide derivatives of the dyes. Glycine betaine titration of these labelled OpuAC mutants (Fig. 3a) was monitored by solution-based alternating laser excitation (ALEX) single-molecule FRET (Fig. 3b). Indeed, we see the FRET signal decreasing in OpuAC (N414C/K566C) and increasing in the other two mutants. The mutant (T504C/K521C) that was predicted to be least surface accessible (8–11%) also showed a glycine-betaine dependent conformational change, however, the apparent dissociation constant (K_D_) of 38 μM is an order of magnitude higher than reported for the wildtype protein and the (D320C/K453C) and (N414C/K566C) mutants^10,20^. Moreover, a low surface accessibility may influence the rotational freedom of the labels. Although we cannot say with certainty that the increased K_D_ of OpuA (T504C/K521C) is due to the labelling of the buried Thr-504, we believe that the RSA is a valuable parameter to restrain in the initial selection of labelling sites. We propose to lower the restraints when the number of pairs is too low, but the labelled protein should always be tested for functionality. The (D320C/K453C) and (N414C/K566C) mutants show K_D_ values in the same range (1–4 μM) as reported for the wildtype protein and were used for further studies.

### Mutations in the full-length transporter OpuA

**Figure 4:**
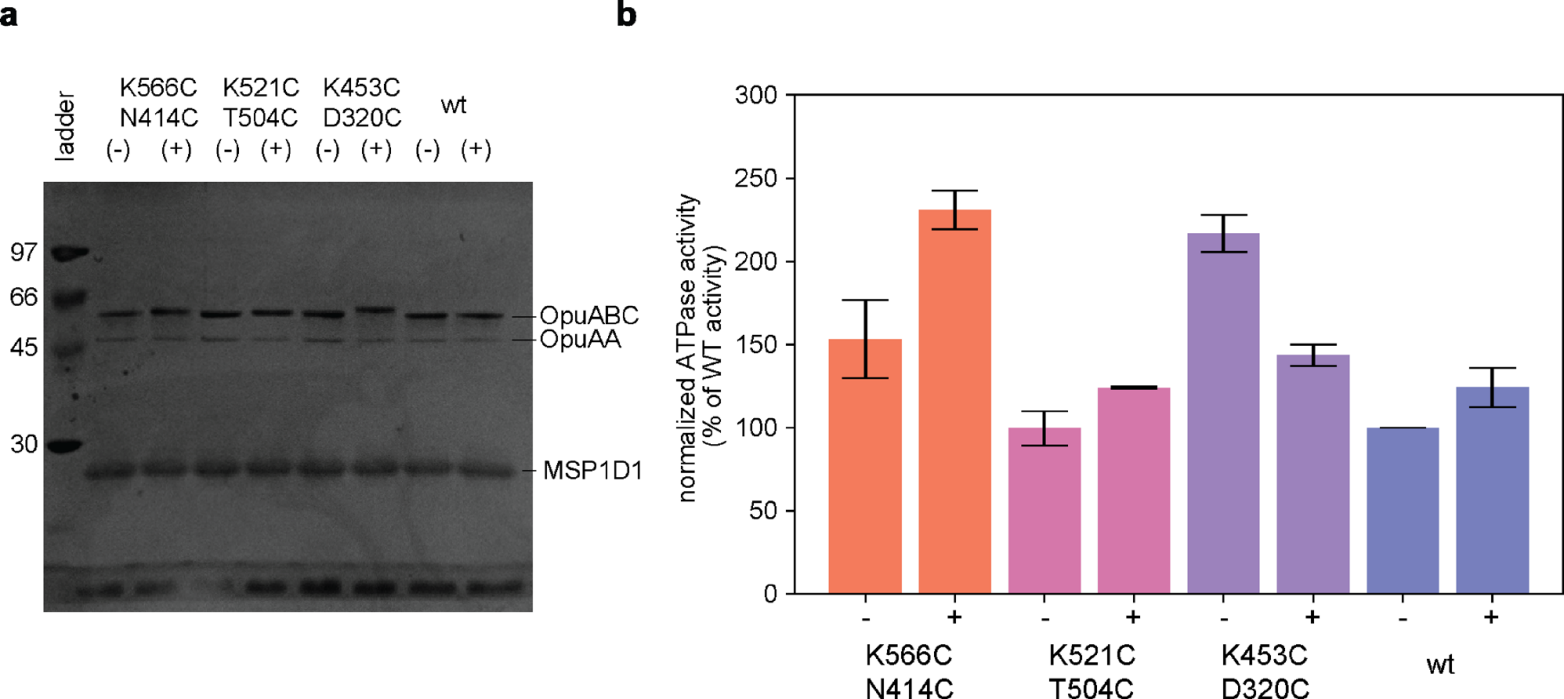
SBD mutations in the full-length transporter (**a**) SDS-PAGE gel showing OpuA nanodiscs of the three mutants and wild-type OpuA before (−) and after (+) labeling with AMdiS (4-acetamido-4′-maleimidylstilbene-2,2′- disulfonic acid) (**b**) Normalized ATPase activity in the presence of 10 mM MgATP, 62 μM glycine betaine and 300 mM KCl of the same nanodiscs as shown in (**a**). The ATPase activity was corrected for the A_280_ absorbance of the nanodiscs (contribution from the OpuA subunits and the MSP1D1 scaffolding protein) and normalized against the activity of unlabeled wild-type OpuA. Datapoints are shown as the average of two technical replicates. Error bars represent the standard deviation.

Next, we verified that the labelling positions do not interfere with the activity of the full-length transporter. OpuA has two SBDs covalently linked to the transmembrane domain, and therefore four cysteines per complex. The three mutant pairs were constructed in the full-length transporter and the proteins were purified and reconstituted in MSP1D1 nanodiscs. After reconstitution, half of the nanodiscs were labelled with 4-acetamido-4′-maleimidylstilbene-2,2′- disulfonic acid (AMdiS) and the other half of the sample was used as control. Like the fluorophores used for smFRET, AmdiS is a relatively bulky water-soluble maleimide but unlike the dyes it is affordable for large-scale protein labelling. We used SDS-PAGE gel electrophoresis to show that the proteins are quantitatively labelled with AmdiS, which is apparent from a significant shift in the migration of the OpuABC subunit of the OpuA complex (Fig. 4a). All fractions were then analysed for ATPase activity using a coupled enzyme assay (Fig. 4b). We do not want to interpret the apparent increase (2 out of 3) or decrease (1 out of 3) in activity upon treatment of the OpuA nanodiscs observed with AMdiS because of uncertainties in the protein concentration. We infer that the glycine betaine-dependent ATP hydrolysis activity of the labelled mutants is comparable to that of the wildtype protein. Since OpuA labelling is better than 90% (unlabelled OpuABC is barely visible in Fig. 4a), we conclude that the OpuA mutants D320C/K453C, T504C/K521C and N414C/K566C are suitable candidates for future studies on the conformational dynamics of OpuA.

## Discussion

We describe a straightforward approach to select sites for labelling of proteins for smFRET or EPR measurements. One can use proteins similar to the one used to showcase the approach, for instance, the receptor or substrate binding domains associated with ABC transporters, tripartite tricarboxylate transporters (TTTs), tripartite ATP-independent periplasmic transporters (TRAP), some ligand-gated ion channels (LGI), metabotropic receptors (GPCRs) or 2-component regulatory systems^23^. In these classes of proteins alone, already in 2016 (last structural classification^23^) there were over 500 structures available. However, in principle, the method is not limited to these proteins, but can be used for any system, provided at least two structures or homology models in different conformations are available.

**Figure 5:**
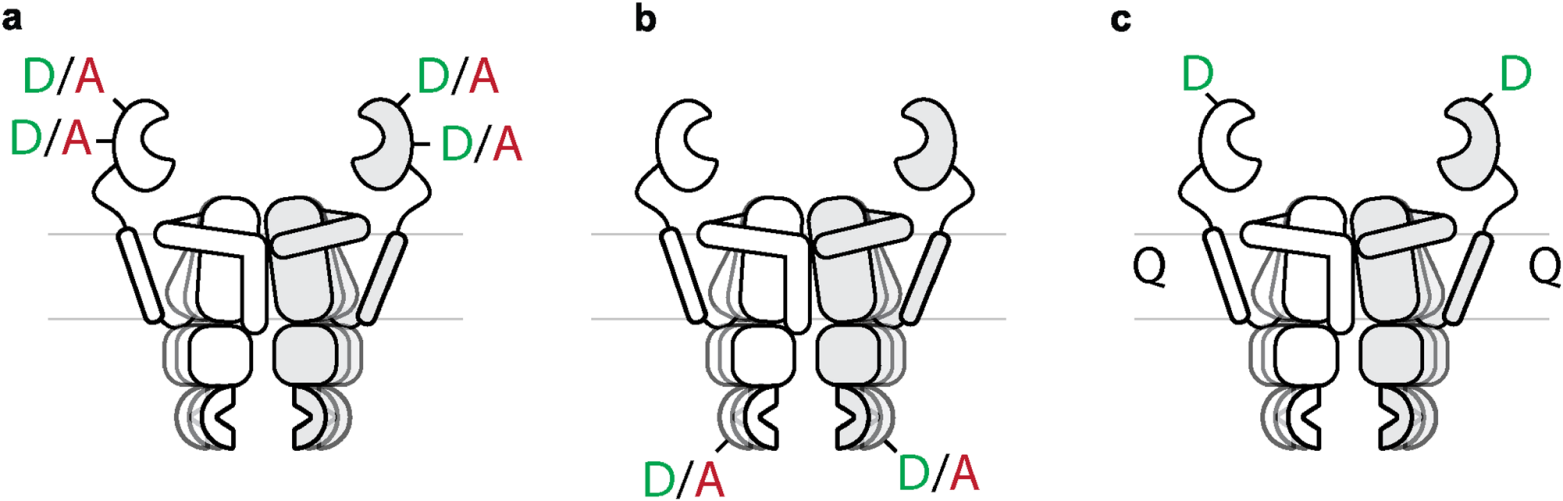
(**a**) Stochastic labeling of a homodimer with 2 cysteine residues per protomer, leading to 2^4^ possible species. (**b**) Stochastic labeling of a homodimer with one cysteine residue per protomer to probe inter-protomer distances. 2^2^ possible species (because the twofold symmetry, both DA and AD species, thus 50% of all species, are available for FRET) (**c**) Labeling of a single cysteine residue per protomer with a fluorescence donor, a fluorescence quencher is added to the lipid bilayer.

Like OpuA, many of the above-mentioned proteins are homodimeric with more than one SBD per functional complex, hence multiple pairs of cysteine residues are present per complex, complicating the smFRET analysis (Fig. 5a). By introducing a single cysteine per domain and stochastic labeling, hence two cysteine residues per complex in case of a homodimeric complex, it will be possible to observe interdomain movements (Fig. 5b), as has been shown for the ABC-transporter BtuCD by labelling the transmembrane domains^24^, the ABC-transporter MRP1 by labelling the NBD’s^25^, but also for the ABC-transporters MsbA^6^ and McjD^7^. A similar approach has been used in smFRET studies on BetP, a homotrimeric protein with three fluorophores per complex^8^. Alternatively, one could label the protein with a fluorescence donor and introduce a fluorescence quencher in the ligand or membrane to probe conformational dynamics. In another study on the ABC transporter BtuCD, the cobalt ion in the ligand (vitamine B_12_) has been used for quenching to determine transfer of the substrate from the SBD through the TMD^9^. By inserting a quencher in for instance the vesicle or nanodisc membrane, and a fluorescence donor in the SBD, one could determine the conditions under which the SBD gets closer to or further away from the membrane (Fig. 5c).

To facilitate smFRET measurements in homodimeric proteins such as OpuA with multiple identical subunits, it should be possible to create apparent heterodimeric complexes with *e.g.* one protomer containing the double cysteine mutation and one protomer being cys-less. One can then probe the opening and closing of the SBD in the context of the full-length transporter and *e.g.* determine if the two SBDs of OpuA deliver substrates stochastically or that a receptor domain once bound can deliver multiple substrates. We aim to take this approach in future studies, building on the work described in this paper. In short, we describe a systematic method to find candidates for FRET, EPR or other double mutation-based distance-reporting methods that can be used to make a pre-selection of suitable pairs using relevant distance and solvent accessibility constraints.

## Material and methods

### Residue selection protocol

For the residue selection protocol, we recommend to follow the instruction of the script (https://doi.org/10.5281/zenodo.4446814). In short: two protein structures (different conformations) are read using the ProDy Python library^26^ Distance maps, containing the distance between the center of mass of the side chain (Cα for glycine) of each pair of residues for each protein structure. The difference map of these two distance maps is generated by subtraction. By selection of distances within a specified range, the amino acid pairs are filtered and only the pairs with a suitable distance (e.g. depending on the probes used for FRET or EPR) between the residues are kept. The DSSP software (version 3.0.0–2)^21,27^ is used to assess the secondary structure and surface accessibility for each of the residues. By filtering based on surface accessibility the number of possible pairs is further reduced. The script returns a list of suitable residue pairs, as well as a script that can be imported in the PyMOL Molecular Graphics System and used for direct visualization of the obtained amino acid pairs.

### Construction of expression strains

The cysteines were introduced sequentially using Quikchange Mutagenesis and the *Escherichia coli* pREOpuAHis vector. Using restriction cloning (*Alw*NI and *Bam*HI), the OpuAC region of the gene, where the mutations were introduced, was transferred into the *Lactococcus lactis* pNZOpuACHis vector, which was used for expression of OpuAC and derivatives The resulting plasmids were transformed into the OpuA deletion strain *L. lactis* Opu401.

### Expression of genes

*L. lactis* Opu401 carrying pNZOpuAHis or pNZOpuACHis were cultivated semi-anaerobically in a 2-L bioreactor at 30 °C, in a rich medium with 2% (w/v) gistex LS (strik BV, Eemnes, NL), 65 mM Potassium phosphate pH 7.0 supplemented with 1% (w/v) glucose and 5 μg mL^−1^ chloroamphenicol. The pH was kept constant at 6.5 by adjusting the medium with 4 M potassium hydroxide. The *nisA* promoter was activated at an OD600 of 2 by adding 0.05% (v/v) of the culture supernatant of the nisin A producing *L. lactis* strain NZ9700^28^. The cells were harvested by centrifugation (15 min, 6000×*g*, 4 °C) after 2 h of induction, washed in 100 mM KPi pH 7.0, centrifuged again (15 min, 6000×*g*, 4 °C), resuspended in ice-cold 100 mM KPi pH 7.0 to a final OD of 100 and stored at − 80 °C until further use.

### Isolation and purification of OpuAC

The cells were lysed by passing them twice through a high-pressure device at 29 k psi (Constant Systems) in the presence of 100 μg mL^−1^ deoxyribonuclease (DNAse) and 2 mM MgSO_4_, followed by the immediate addition of 5 mM Na_2_-EDTA (pH 8.0), 1 mM PMSF plus 1 mM DTT for the cysteine mutants after breaking. Cell debris and membrane vesicles were removed by centrifugation (90 min; 125,000×*g*; 4 °C). The cell lysate was aliquoted in samples of 20 mL and flash-frozen in liquid nitrogen and kept at − 80 °C until further use. 1 mL column volume of Ni^2+^-Sepharose resin was equilibrated with 12 column volumes of water and 2 column volumes of 50 mM potassium phosphate pH 7.0, 200 mM KCl, 15 mM imidazole and 1 mM DTT. 40 mL of cell lysate was thawed rapidly and supplemented with 10 mM imidazole, 1 mM DTT and the equilibrated resin. The column was poured, drained, and washed with 20 column volumes of 50 mM potassium phosphate pH 7.0, 200 mM KCl plus 1 mM DTT. The protein was eluted in 0.6 column volumes of elution buffer (50 mM potassium phosphate pH 7.0, 200 mM KCl, 500 mM imidazole plus 1 mM DTT) for the first fraction and 0.4 column volumes of the same buffer for the later fractions.

### Labeling of OpuAC for single-molecule FRET

100 μL column volume of Ni^2+^-Sepharose resin was equilibrated with 1–2 column volumes of water and 1–2 column volumes of 50 mM potassium phosphate pH 8.0, 200 mM KCl and 1 mM DTT. 10 nmol of unlabelled OpuAC was added to the column and allowed to bind for 1 h at 4 °C. Then, the column was washed with 10 volumes of 50 mM potassium phosphate pH 8.0, 200 mM KCl to remove the DTT. The column was closed, and 1 mL of the same buffer was added. Next, 50 nmol of Alexa555 and Alexa647 were dissolved in 10 μL water free DMSO and added to the column. The labelling reaction was performed overnight at 4 °C. The next day, the column was washed with 10 column volumes of 50 mM potassium phosphate pH 8.0, 200 mM KCl and eluted in 700 μL of the same buffer supplemented with 500 mM imidazole.

### Isolation and purification of OpuA

The cells were lysed by passing them twice through a high-pressure device at 29 k psi (Constant Systems) in the presence of 100 μg mL^−1^ deoxyribonuclease (DNAse) and 2 mM MgSO4, followed by the immediate addition of 5 mM Na_2_-EDTA (pH 8.0), 1 mM PMSF plus 1 mM DTT for the cysteine mutants after breaking. Cell debris was removed by centrifugation (15 min; 22,000×*g*; 4 °C), after which the membranes were harvested in an ultracentrifugation step (90 min; 125,000×*g*; 4 °C). The membranes were resuspended in 50 mM KPi pH 7.0 supplemented with 20% (v/v)w/v) glycerol plus 1 mM DTT for the cysteine mutants to a total protein concentration of 10 mg mL^−1^, flash-frozen in liquid nitrogen and kept at − 80 °C until further use. 0.5 mL column volume of Ni^2+^-Sepharose resin was equilibrated with 12 column volumes of water and 2 column volumes of 50 mM potassium phosphate pH 7.0, 200 mM KCl, 15 mM imidazole and 0.02% DDM. Membrane vesicles containing OpuA were thawed quickly and diluted to a total protein concentration of 3 mg mL^−1^. The solubilization of OpuA was performed at 4 °C with 0.5% DDM for 60 min, followed by a centrifugation step (20 min, 270,000×*g*, 4 °C) to remove the insoluble material. The supernatant was diluted 2.5 times and added to the equilibrated resin, after which they were incubated for 1 h at 4 °C. The column was poured, drained, and washed with 20 column volumes of 50 mM potassium phosphate pH 7.0, 200 mM KCl, 20% glycerol, 50 mM imidazole plus 0.02% DDM. OpuA was eluted in 0.6 column volumes of elution buffer [50 mM potassium phosphate pH 7.0, 200 mMKCl, 20% (v/v)w/v) glycerol, 500 mM imidazole plus 0.02% DDM] for the first fraction and 0.4 column volumes of the same buffer for the later fractions. The obtained protein was used immediately for reconstitution in nanodiscs.

### Reconstitution of OpuA in MSP1D1 nanodiscs

The reconstitution procedure was similar to^29^. In short: 1.4 μM of the purified OpuA was mixed with 14 μM purified MSP1D1 scaffold protein and 1.4 mM lipids (lipid composition: 50% DOPE, 12% DOPC, 38% DOPG) in 50 mM potassium phosphate pH 7.0, 4% (v/v) glycerol, 10 mM DDM plus 1 mM DTT to a total volume of 2 mL and was nutated for an hour at 4 °C. Then 2 g of SM2-Biobeads (Bio-rad) were added to adsorb the detergent and this mixture was allowed to incubate overnight. In the morning the supernatant was separated from the Biobeads with a syringe.

### Labeling of OpuA for ATPase assay

Two times 200 μL column volume of Ni^2+^-Sepharose resin was equilibrated with 1 to 2 column volumes of water and 1–2 column volumes of 50 mM potassium phosphate pH 8.0, 200 mM KCl and 1 mM DTT. The reconstitution mixture was split in two samples of 2 mL. The mixture was let to bind to the column (1 hr 4 °C), which then was washed with 10–20 column volumes of buffer without DTT (50 mM potassium phosphate pH 8.0 plus 200 mM KCl). Then, 1 mL of the same buffer supplemented with 1 mM AMdiS (4-acetamido-4′- maleimidylstilbene-2,2′- disulfonic acid) was added and let to react for 1 h at 4 °C. The columns were washed with 10 to 20 column volumes of 50 mM potassium phosphate pH 8.0, 200 mM KCl plus 1 mM DTT. 500 μL of the same buffer supplemented with 500 mM imidazole was added, the columns were closed and left O/N. The next day the elution was collected and immediately used for the ATPase activity assay.

### ATPase activity assays

As described in detail in^29^, we used a coupled enzyme assay with pyruvate kinase (PK) and lactate dehydrogenase (LDH) to determine the ATPase activity of OpuA, which is stoichiometrically coupled to the NADH absorbance decrease at 340 nm. The enzymes PK and LDH were present in excess over OpuA in terms of activity. The NADH absorbance was followed in 96-well plates using a Tecan Spark 10 m plate reader. Each well contains 50 mM KPi pH 7, 300 mM KCl, 4 mM phosphoenolpyruvate, 62 μM glycine betaine, 300 μM NADH, 2.1–3.5 units of pyruvate kinase and 3.2–4.9 units of lactate dehydrogenase. The wells were then supplemented with 100 μL of the elution fraction after labelling, corresponding to roughly 1–5 μM of OpuA. The reaction was started by the addition of 10 mM Mg ATP.

### Single-molecule FRET

Solution-based smFRET and alternating laser excitation (ALEX) experiments were carried out at 5–25 pM of labelled protein at room temperature in 50 mM KPi pH 7.0 supplemented with 1 mM Trolox and 10 mM MEA for photo stabilization plus the reported glycine betaine concentrations. Microscope cover slides (no. 1.5H precision cover slides, VWR Marienfeld) were coated with 1 mg/mL of BSA for 30–60 s to prevent fluorophore and/or protein interactions with the glass material. The excess BSA was then removed by washing and exchanged with 50 mM KPi pH 7.0. All smFRET experiments were performed with a home-built confocal microscope. In brief, two laser-diodes (Coherent Obis) with emission wavelength of 532 and 637 nm were directly modulated for alternating periods of 50 μs and used for confocal excitation. The laser beams were coupled into a single-mode fibre (PM-S405-XP, Thorlabs) and collimated (MB06, Q-Optics/Linos) before entering an oil immersion objective (60X, NA 1.35, UPlanSAPO 60XO, Olympus). The fluorescence was collected by excitation at a depth of 20 μm. Average laser powers were 30 μW at 532 nm (30 kW/cm^2^) and 15 μW at 637 nm (15 kW/cm^2^). Excitation and emission light were separated by a dichroic beam splitter (zt532/642rpc, AHF Analysentechnik), which is mounted in an inverse microscope body (IX71, Olympus). Emitted light was focused onto a 50 μm pinhole and spectrally separated (640DCXR, AHF Analysentechnik) onto two single-photon avalanche diodes (TAU-SPADs-100, Picoquant) with appropriate spectral filtering (donor channel: HC582/75; acceptor channel: Edge Basic 647LP; AHF Analysentechnik). Registration of photon arrival times and alternation of the lasers was controlled by an NI-Card (PXI-6602, National Instruments). Analysis of the photon arrival times were done as described before^10^. In short, a ‘dual channel burst search’^30^ was used to identify fluorescence bursts. The NDA (acceptor emission upon donor excitation), NDD (donor emission upon donor excitation) and NAA (acceptor emission upon acceptor excitation) photocounts were measured per burst and assignments are based on the excitation period and detection channel^31^. The photon counts were corrected for background, where the background counts were estimated by calculating the mean count rate over all bins with more than 20 counts. The apparent FRET efficiency was calculated as NDA/(NDA + NDD) and the Stoichiometry S by (NDA + NDD)/(NDA + NDD + NAA)^24^. Binning the detected bursts into 2D histograms with the apparent-FRET versus Stoichiometry allowed the selection of the donor and acceptor labelled molecules and reduce fluorophore bleaching artifacts^31^. The selected 1D apparent-FRET histograms were fitted, using the method of least squares, with a Gaussian distribution, yielding a 95% confidence interval for the mean.

## References

[1] Kühlbrandt W (2014). The resolution revolution. Science.

[2] Nakane T (2020). Single-particle cryo-EM at atomic resolution. Biorxiv.

[3] Pandey S (2020). Time-resolved serial femtosecond crystallography at the European XFEL. Nat. Methods.

[4] Hofmann S (2019). Conformation space of a heterodimeric ABC exporter under turnover conditions. Nature.

[5] Reginsson GW, Schiemann O (2011). Pulsed electron-electron double resonance: Beyond nanometre distance measurements on biomacromolecules. Biochem. J..

[6] Liu Y, Liu Y, He L, Zhao Y, Zhang XC (2018). Single-molecule fluorescence studies on the conformational change of the ABC transporter MsbA. Biophys. Rep..

[7] Husada F (2018). Conformational dynamics of the ABC transporter McjD seen by single-molecule FRET. EMBO J..

[8] Jazi AA (2017). Caging and photoactivation in single-molecule förster resonance energy transfer experiments. Biochemistry.

[9] Goudsmits JMH, Slotboom DJ, Van Oijen AM (2017). Single-molecule visualization of conformational changes and substrate transport in the Vitamin B12 ABC importer BtuCD-F. Nat. Commun..

[10] de Boer M (2019). Conformational and dynamic plasticity in substrate-binding proteins underlies selective transport in ABC importers. eLife.

[11] Nanda JS, Lorsch JR (2014). Labeling of a protein with fluorophores using maleimide derivitization. Methods Enzymol..

[12] Joseph B (2019). In situ observation of conformational dynamics and protein ligand–substrate interactions in outer-membrane proteins with DEER/PELDOR spectroscopy. Nat. Protoc..

[13] Halbmair K, Wegner J, Diederichsen U, Bennati M (2016). Pulse EPR measurements of intramolecular distances in a TOPP-labeled transmembrane peptide in lipids. Biophys. J..

[14] Wood E (1994). Molecular probes: Handbook of fluorescent probes and research chemicals. Biochem. Educ..

[15] Tsvetkov YD, Bowman MK, Grishin YA (2019). Pulsed Electron-Electron Double Resonance. Pulsed Electron-Electron Double Resonance.

[16] Margeat E (2006). Direct observation of abortive initiation and promoter escape within single immobilized transcription complexes. Biophys. J..

[17] Lee NK (2005). Accurate FRET measurements within single diffusing biomolecules using alternating-laser excitation. Biophys. J..

[18] Müller BK, Zaychikov E, Bräuchle C, Lamb DC (2005). Pulsed interleaved excitation. Biophys. J..

[19] Sikkema HR (2020). Gating by ionic strength and safety check by cyclic-di-AMP in the ABC transporter OpuA. Sci. Adv..

[20] Wolters JC (2010). Ligand binding and crystal structures of the substrate-binding domain of the ABC transporter OpuA. PLoS ONE.

[21] Kabsch W, Sander C (1983). Dictionary of protein secondary structure: Pattern recognition of hydrogen-bonded and geometrical features. Biopolymers.

[22] Tien MZ, Meyer AG, Sydykova DK, Spielman SJ, Wilke CO (2013). Maximum allowed solvent accessibilites of residues in proteins. PLoS ONE.

[23] Scheepers GH, Lycklama a Nijeholt JA, Poolman B (2016). An updated structural classification of substrate-binding proteins. FEBS Lett..

[24] Yang M (2018). Single-molecule probing of the conformational homogeneity of the ABC transporter BtuCD. Nat. Chem. Biol..

[25] Wang L (2020). Characterization of the kinetic cycle of an ABC transporter by single-molecule and cryo-EM analyses. eLife.

[26] Bakan A, Meireles LM, Bahar I (2011). ProDy: Protein dynamics inferred from theory and experiments. Bioinformatics.

[27] Joosten RP (2011). A series of PDB related databases for everyday needs. Nucleic Acids Res..

[28] Kuipers OP, de Ruyter PGGA, Kleerebezem M, de Vos WM (1998). Quorum sensing-controlled gene expression in lactic acid bacteria. J. Biotechnol..

[29] Karasawa A (2013). Physicochemical factors controlling the activity and energy coupling of an ionic strength-gated ATP-binding cassette (ABC) transporter. J. Biol. Chem..

[30] Nir E (2006). Shot-noise limited single-molecule FRET histograms: Comparison between theory and experiments. J. Phys. Chem. B.

[31] Kapanidis AN (2004). Fluorescence-aided molecule sorting: Analysis of structure and interactions by alternating-laser excitation of single molecules. Proc. Natl. Acad. Sci. USA.

